# Top 100 most-cited publications in hidradenitis suppurativa: An updated bibliometric analysis

**DOI:** 10.3389/fmed.2022.995873

**Published:** 2022-09-08

**Authors:** Yan Teng, Sujing Li, Yibin Fan, Xiaohua Tao, Youming Huang

**Affiliations:** ^1^Department of Dermatology, Health Management Center, Zhejiang Provincial People’s Hospital, Affiliated People’s Hospital, Hangzhou Medical College, Hangzhou, China; ^2^Bengbu Medical College, Bengbu, China

**Keywords:** hidradenitis suppurativa, bibliometric analysis, citation, web of science, most-cited publications

## Abstract

**Background:**

Over the last several decades, our understanding of hidradenitis suppurativa (HS) has improved considerably, thereby enhancing our ability to clinically diagnose and treat the disease.

**Objective:**

The purpose of this study was to identify and analyze the top 100 most-cited publications related to HS to update bibliometric information on HS.

**Materials and methods:**

We used the Web of Science database to identify reports on hidradenitis suppurativa. Data from the 100 most-cited publications were extracted and analyzed.

**Results:**

The citation number of the top 100 most-cited articles was 89–532 (mean, 153.51), with the most productive periods being from years 2007 to 2016. Most publications originated from the *British Journal of Dermatology* and the *Journal of the American Academy of Dermatology*. The 100 articles originated from 18 countries, with Denmark being the most productive country, followed by the United States (17), England (14), and Germany (12). Jemec GB, from the University of Copenhagen, had 32 citations and was the most frequently identified author. The 100 articles encompassed several fields of research as follows: pathogenesis (18%), pathophysiology (7%), epidemiology (14%), clinical diagnosis and features (16%), treatment (25%), comorbidity (10%), and others (10%). In total, 11 reviews, three guidelines, and 86 original articles (nine randomized clinical trials) were included.

**Conclusion:**

Through this bibliometric analysis, we aimed to indicate a series of intellectual landmark publications that offer us critical reviews, guidelines, and original articles, which highlight the immense level of progress achieved in the field of HS.

## Introduction

Hidradenitis suppurativa (HS), also known as acne inversa, is a chronic, inflammatory, and debilitating cutaneous disorder. This condition is characterized by the presence of multiple types of skin lesions, including inflammatory nodules, abscesses, sinus tract, and rope-like scarring, which may appear simultaneously on one patient. HS prevalence is considerably influenced by the method of data collection. Therefore, the exact incidence rate of HS remains unknown. The name “hidradenitis suppurativa” suggests that it is a suppurative disease primarily involving sweat glands. However, improvements in our understanding of the associated pathogenesis have helped researchers establish the prevailing theory that HS is a chronic follicular occlusive disease that primarily involves the follicular portion of folliculopilosebaceous units (1, 2). Follicular obstruction, follicular rupture, and an associated immune response appear to be key events in the occurrence and development of HS clinical manifestations (3). Recurring lesions and associated pain, discomfort, and purulent and malodorous discharge that accompany HS exert a profound psychosocial influence on many patients (4). Notably, over the last several decades, our understanding of HS, including the pathogenesis, clinical manifestations, diagnosis, and treatment, has improved considerably, which has subsequently enhanced our ability to manage the condition.

Since the beginning of the 20th century, a large number of studies have attempted to provide new insights into HS, and numerous articles—including reviews, guidelines, and original articles, among others—related to HS have emerged. Citation analysis is used as a quantitative and bibliometric method to determine the frequency and pattern of citations in specific scientific fields. Citation analysis can be applied to obtain an objective measure to evaluate current research interests and the scientific impact of pertinent published achievements. Frequently cited articles that identify important new findings and trends in a scientific field can be screened through citation analysis, and the outcomes can be used to indicate the development of a specific discipline (5). Various fields in dermatology like psoriasis arthritis (6), rosacea (7), melanoma (8), and Meckel cell carcinoma (9) have been thoroughly investigated through citation bibliometric analysis. Seivright et al. (10) performed a bibliometric analysis of the top 50 most-cited publications in 2020, whereas, 2 years later, we performed a similar study to obtain more comprehensive knowledge of the influential studies on HS. The purpose of the present study was to identify the 100 top most-cited publications in HS to highlight the most significant advances in the field over the past several decades. This knowledge can be used to better understand the classical studies that have significantly contributed to the field of HS.

## Materials and methods

### Searching strategy

On 14 May 2022, a search of the Web of Science database^1^ was performed with the search phrase “hidradenitis suppurativa” or “acne inversa” or “Verneuil’s disease.” No limitation was established with regard to the data range. Articles were ranked according to their citation numbers in descending order. The articles with the same citation number were ranked according to date of publication, with the more recent articles ranked relatively higher. Two of the listed authors (Yan Teng and Sujing Li) independently screened each article through the title and abstract. Reports that had HS as the main focus of the research were included. The articles that had HS as part of a group of diseases and focused only on other topics were excluded; we also excluded conference articles, case reports, and controversies. The two researchers eventually achieved an agreement on the list of the 100 most-cited publications after the validation of senior dermatology experts (Xiaohua Tao and Youming Huang). Animal experiments or clinical studies were not included, thereby waiving the need for approval from an ethical committee.

### Data extraction and statistical analysis

The list of the top 100 most-cited publications on HS was extracted to Microsoft Excel 2021. Data information including study title, author names, journal name, year of publication, citation number, and country of origin were directly obtained from the search results. Further assessments were performed to classify the reports according to the following article types: original articles (RCTs included), reviews, and guidelines. The research topics were determined by Yan Teng and Sujing Li by evaluating the title, abstract, and keywords of the 100 publications. Journal impact factors were included in the 2021 Incites Journal Citation Reports. All figures were examined using Microsoft Excel 2021.

The top 100 cited publications were selected to be analyzed further, and the information of study title, author listing, journal name, year, citation number, and country of origin was recorded.

## Results

The 4,820 results were sorted in descending order according to the citation number. The information on the 100 most-cited publications is listed in Supplementary Table 1.

### Year of publication

**FIGURE 1:**
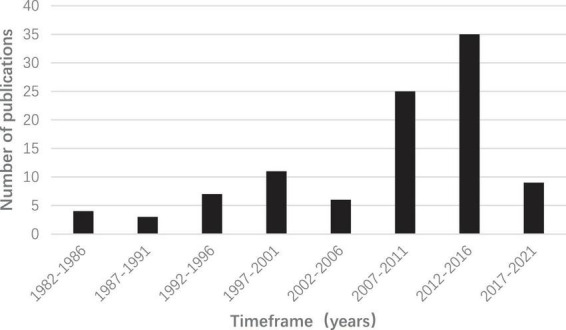
A bar graph showing the time frame of publication and number of publications per time frame for the top 100 most-cited reports on hidradenitis suppurativa.

The 100 most-cited articles were published between the years 1983 and 2020; however, 60 articles were published in the most productive periods, i.e., 2007–2011 and 2012–2016 (Figure 1). The highest number of articles published in a single interval was 35, and these reports had been published in 2012–2016. The oldest of these publications, “Topical treatment of hidradenitis suppurativa with clindamycin” by Clemmensen, was published in 1983. The most recent of these articles is a review, and its title is “Hidradenitis suppurativa,” published by Sabat et al. in 2020.

### Citations

The top 100 most-cited articles on HS were cited 89 (article numbers 100) to 532 (top article) times, with 15,351 citations (mean, 153.51 citations per article). Among these publications, “European S1 guideline for the treatment of hidradenitis suppurativa/acne inversa” by Zouboulis et al. was the most-cited article.

### Journal of publications

In total, 27 different journals published the 100 most-cited publications, with the *British Journal of Dermatology* contributing most studies (*n* = 32), followed by *Journal of the American Academy of Dermatology* (*n* = 21), *JAMA Dermatology* (*n* = 6), *Journal of the European Academy of Dermatology and Venereology* (*n* = 5), *Acta Dermato-Venereologica* and *Dermatology* (*n* = 4), and *the Journal of investigative dermatology* (*n* = 3). The remaining journals each published less than three articles on the list presented in Table 1.

**TABLE 1:** Journal names for the top 100 most-cited publications in hidradenitis suppurativa stratified by number of articles in the top in descending order; impact factors from 2021 are also recorded.

Journal	Number of articles	Impact factor
*British Journal of Dermatology*	32	11.113
*Journal of the American Academy of Dermatology*	21	15.487
*JAMA Dermatology*	6	11.816
*Journal of the European Academy of Dermatology and Venereology*	5	9.228
*Dermatology*	4	5.197
*Acta Dermato-Venereologica*	4	3.875
*The Journal of Investigative Dermatology*	3	7.590
*Experimental Dermatology*	2	4.511
*Archives of Dermatology*	2	NA
British Medical Journal	2	93.333
Dermatologic Surgery	2	2.914
International Journal of Dermatology	2	3.204
New England Journal of Medicine	2	176.079
American Journal of Clinical Dermatology	1	6.233
Annals of Internal Medicine	1	51.598
British Journal of Plastic surgery	1	NA
Clinical and Experimental Dermatology	1	4.481
Inflammatory Bowel Diseases	1	7.29
International Journal of Colorectal Disease	1	2.796
JAMA	1	157.335
Nature Reviews. Disease Primers	1	65.038
PloS One	1	3.752
Reviews in Endocrine and Metabolic Disorders	1	9.306
Science	1	63.714
Clinical, Cosmetic, and Investigational Dermatology	1	2.765
The Journal of Immunology	1	5.426

### Countries, authors, institutes, and departments

**FIGURE 2:**
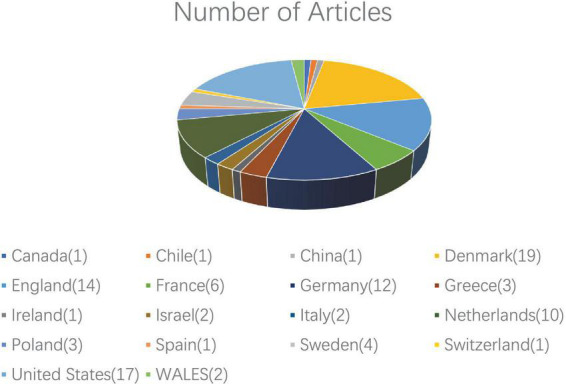
The top 100 most-cited articles on hidradenitis suppurativa according to country.

Overall, the 100 articles originated from 18 countries. The country with the largest number of articles was Denmark (*n* = 19), followed by the United States (*n* = 17), England (*n* = 14), Germany (*n* = 12), Netherlands (*n* = 10), and France (*n* = 7) (Figure 2).

Four authors have published ≥ 10 highly cited HS reports. This list included Jemec (*n* = 29, Roskilde Hospital, University of Copenhagen), Prens (*n* = 16, Erasmus, University Medical Center), van der Zee (*n* = 12, Erasmus, University Medical Center), and Boer (*n* = 10, Deventer Hospital).

### Article type and research focus

The top 100 most-cited articles reported studies on the following fields of research: pathogenesis (18%), pathophysiology (7%), epidemiology (14%), clinical features and diagnosis (16%), treatment (25%), comorbidity (10%), and others (10%). Eleven reviews, three guidelines, and 86 original articles were included. In the 100 articles, there were nine randomized clinical trials (RCTs) on the treatment, of these five focused on the administration of TNF-a inhibitors (three on adalimumab, one on infliximab, and one on etanercept), and the other four, respectively, focused on the treatment of topical clindamycin versus systemic tetracycline, cyproterone acetate, topical clindamycin, and Anakinra—a type of IL-1 receptor antagonist. Regarding triggering factors of HS, eight articles highlighted that smoking and overweight were important factors that induced or exacerbated the course of the disease. Additionally, metabolic syndrome and psychiatric comorbidities are highlighted in a relatively large proportion of articles on comorbidities of HS.

## Discussion

Although HS was first described 200 years ago, it has been underestimated as a disease for a long period. The specific pathogenesis and effective treatment methods have not been elucidated thus far. In the present study, we aimed to update the bibliometric citations analysis in HS by identifying and analyzing the top 100 most-cited publications.

The included articles were published between 1983 and 2020. Our analysis revealed that most of the top-cited articles on HS were published after 1997, with the largest subset consisting of studies published between 2012 and 2016. This trend might be attributable to the improvement of diagnostic and treatment levels for HS. Before 1997, the highest number of publications was observed in 1996. There are six articles with a total citation number of 852. These articles clarified HS from various perspectives, including epidemiology, pathophysiology, pathogenesis, and clinical diagnosis, thereby providing a suitable foundation for a large number of investigations on HS.

Hidradenitis suppurativa (HS) diagnosis primarily depends on the typical lesions and the disease course. However, histopathological examination is an important diagnostic measure and contributes to the investigation of pathogenesis and pathophysiology. An article entitled “*Histology of hidradenitis suppurativa*” clarified that the HS is primarily involved in the follicular portions, and the involvement of apocrine glands appears only in a minority of axillary lesions (11). In the article entitled “*Hidradenitis suppurativa/acne inversa: bilocated epithelial hyperplasia with very different sequelae*,” von Laffert et al. (12) indicated that the histopathologic manifestations of HS vary, depending on the stage of the lesion. Follicular hyperkeratosis, follicular plugging, follicular dilation, and lymphocytic perifolliculitis belong to the common early features. Biopsies of established areas of disease can demonstrate other features, including psoriasiform hyperplasia of the interfollicular epithelium or a dense, mixed inflammatory infiltrate involving the lower half of the dermis and subcutis. The first most-cited article titled “*European S1 guideline for the treatment of hidradenitis suppurativa/acne inversa*” was published in 2015, with a total citation number of 532, whereas the total citation number indicated by Seivright et al. (10) was 402, which ranked two in the top 50 most-cited articles. The comparison demonstrated the rapid rise in the citation number for this publication. This trend could be explained by the fact that the European S1 HS guideline provided a comprehensive presentation on definition, clinical presentation, epidemiology, pathogenesis, complications, and various therapies (13).

The results of our study revealed that the great majority of the 100 top-cited HS articles were published in the *British Journal of Dermatology* and *Journal of the American Academy of Dermatology*, both of which are extremely well-known in the field of dermatology. This might reflect the inherent bias of researchers to select high-impact journals for submission and citation. Publications in the last 5 years (2016–2020) were published in eight different journals. The fact might demonstrate the increasing interest in the research of HS, owing to the in-depth understanding of its pathogenesis and treatment. The author Jemec from Germany contributed the most among the 100 top-cited articles, with 29 publications under his belt. His studies referred to various aspects of HS, including the natural history, epidemiology, pathogenesis, diagnosis, and treatment. Additionally, Prens and van der Zee HH, with the same origin as that of the Department of Dermatology, Erasmus MC, University Medical Center, both contributed considerably to the list presented in the current study. Researchers worldwide contributed to the HS research, especially in Denmark, England, and the United States, which might be explained by the relatively high prevalence and, more importantly, the level of economic development in the abovementioned countries. Despite this, the quality of the article is extremely critical for the high citations instead of countries, institutes, or authors.

In this study, we also analyzed the article type and research focus. The majority of the publications were original articles, followed by reviews that systematically summarized the recent advances in the field of HS. The article with the highest number of citations was a review, which may be attributable to the fact that review articles were frequently cited and always accepted by journals with a relatively higher impact factor. The pathogenesis of HS ranked first in the research focus.

In brief, the occurrence of HS is multifactorial and is associated with interplay between genetic, environmental, lifestyle, and endocrine factors, in addition to microbiota, that trigger the immune response around the hair follicles. The relatively important triggers highlighted in the most-cited articles are smoking and overweight. Eight articles highlighted the association between obesity and HS, which may be due to the presence of relatively larger intertriginous areas, local skin irritation caused by sweat retention, narrowing follicular orifices following intrafollicular keratin hydration during skin occlusion, and increased levels of circulating pro-inflammatory cytokines associated with obesity. In addition, similar to acne vulgaris, hormonal changes associated with obesity may contribute to the relative androgen overproduction and are proposed to increase follicular plugging, as one aspect of the pathogenesis (14–16). The strong relationship between smoking and HS has been clarified in articles 5 and 30. This might be attributable to the combined effects of the following factors: nicotine and other components of tobacco on follicular occlusion, neutrophil chemotaxis, TNF-a production by keratinocytes, and Th17 cells (17–19). However, long-standing, poorly controlled HS could be responsible for causing significant physical and mental comorbidities. Furthermore, our analysis revealed that four articles clarified the relationship between metabolic syndrome and HS. These findings indicated that patients with HS may have an increased risk for certain diseases, such as metabolic syndromes and associated comorbidities, a range of metabolic and secondary disorders, including diabetes, obesity, insulin resistance, dyslipidemia, hyperglycemia, hypertension, myocardial infarction, and cerebrovascular accidents (20–23). Depression is also an important and common comorbidity, as highlighted in article 31 (24). This article reported that, in comparison with other dermatological disorders, HS has a severe impact on the quality of life. The Major Depression Inventory of patients with HS correlates with the severity of the disease. Besides, among the 100 top-cited articles, the articles on the treatment of HS accounted for a large proportion. RCTs were all from the treatment group. Except for the traditional therapeutic methods, such as antibiotics [clindamycin, tetracycline, and tetracycline (25), isotretinoin (26), anti-androgen therapy with cyproterone acetate (27), and metformin (28), the function of biologic agents to treat HS has been clarified. These biologic agents include TNF-a inhibitors, including etanercept, adalimumab, and infliximab; interleukin-1 (IL-1) receptor antagonist-like Anakinra; and an anti-IL/12/IL-23 antibody, such as Ustekinumab (29–33). Surgical excision has always been an indispensable therapy to manage the chronic refractory lesions of HS, and this point has been clarified in three articles (34–36). As highlighted by the European guidelines, the treatment of HS should be based on the assessment of the inflammatory factors and scarring, and should be administered according to evidence-based guidelines (37).

Owing to the fact that bibliometric studies are widely applied in various fields of research, the citation number could not reflect the true influence of the articles. This may be affected by several factors, including the time of publication, the origin of authors, and the impact of the journals. Additionally, the articles could be cited one time to be praised or criticized, thereby increasing the citation rate of the article. Therefore, we need to treat the most-cited articles from a dialectical perspective.

## Conclusion

Our study offered an updated and comprehensive bibliometric analysis of the top 100 most-cited publications on HS. These articles were published between 1983 and 2000. The focus on the pathogenesis and treatment of HS was highlighted in most of the articles. Biologic agents are, currently, the cutting-edge and effective treatment methods. Additionally, physical and mental comorbidities, including metabolic syndrome and depression, are among the highlighted research focuses. The 100 articles originated from 18 different countries, mostly in Denmark and the United States. To the best of our knowledge, this may be the latest and relatively comprehensive study to identify the classic articles on the topic of HS and provide clinical physicians and researchers with valuable information to establish clinical decisions and perform investigations in the future.

## Data availability statement

The original contributions presented in this study are included in the article/Supplementary material, further inquiries can be directed to the corresponding authors.

## Author contributions

YT: study concepts. YH and XT: study design. YF: literature research. YT and SL: manuscript preparation and editing. All authors contributed to the article and approved the submitted version.

## Footnotes

1
https://www.webofscience.com/wos/woscc/basic-search
