# Supplementary material for: Top 100 most-cited publications in hidradenitis suppurativa: An updated bibliometric analysis
Source: Front Med (Lausanne). 2022 Sep 8;9:995873. doi: 10.3389/fmed.2022.995873 (PMC9493351; doi:10.3389/fmed.2022.995873)
Supplement: Supplementary file 1 [file Table_1.pdf]

Supplementary Table 1 The top 100 Most-cited Articles on hidradenitis suppurativa are organized by citation number in descending order. Article types are as follows: O (original articles), RCT (Randomized Control Trials), R (Reviews), and G (Guidelines).

Rank	First author	Year	Title	Journal	Citations	Type
1	Zouboulis CC	2015	European S1 guideline for the treatment of hidradenitis suppurativa/acne inversa	*Journal of the European Academy of Dermatology and Venereology*	532	G
2	Jemec GBE	2012	Hidradenitis Suppurativa	*New England Journal of Medicine*	487	R
3	Alikhan A	2009	Hidradenitis suppurativa: A comprehensive review	*Journal of the American Academy of Dermatology*	401	R
4	Revuz JE	2008	Prevalence and factors associated with hidradenitis suppurativa: results from two case-control studies	*Journal of the American Academy of Dermatology*	393	O
5	Sartorius K	2009	Objective scoring of hidradenitis suppurativa reflecting the role of tobacco smoking and obesity	*British Journal of Dermatology*	326	O
6	Kimball AB	2016	Two phase 3 trials of adalimumab for hidradenitis suppurativa	*New England Journal of Medicine*	302	RCT
6	Van der Zee HH	2011	Elevated levels of tumour necrosis factor (TNF)-alpha, interleukin (IL)-1 beta and IL-10 in hidradenitis suppurativa skin: a rationale for targeting TNF-alpha and IL-1 beta	*British Journal of Dermatology*	301	O
7	Jemec GB	1996	The prevalence of hidradenitis suppurativa and its potential precursor lesions	*Journal of the American Academy of Dermatology*	298	O
8	Kimball AB	2012	Adalimumab for the treatment of moderate to severe hidradenitis suppurativa: a parallel randomized trial	*Annals of Internal Medicine*	264	RCT

9	Grant A	2010	Infliximab therapy for patients with moderate to severe hidradenitis suppurativa: a randomized, double-blind, placebo-controlled crossover trial	*Journal of the American Academy of Dermatology*	264	RCT
10	Wang B	2010	γ-secretase gene mutations in familial acne inversa	*Science*	259	O
11	Wolkenstein P	2007	Quality of life impairment in hidradenitis suppurativa: A study of 61 cases	*Journal of the American Academy of Dermatology*	258	O
12	Vazquez BG	2013	Incidence of hidradenitis suppurativa and associated factors: a population-based study of Olmsted County, Minnesota	*Journal of Investigative Dermatology*	242	O
13	Revuz J	2010	Hidradenitis suppurativa	*Journal of the European Academy of Dermatology and Venereology*	234	R
14	Schlapbach C	2011	Expression of the IL-23/Th17 pathway in lesions of hidradenitis suppurativa	*Journal of the American Academy of Dermatology*	228	O
15	Von der Werth JM	2001	Morbidity in patients with hidradenitis suppurativa	*British Journal of Dermatology*	221	O
16	Matusiak Ł	2010	Psychophysical aspects of hidradenitis suppurativa	*Acta Dermato-Venereologica*	206	O
17	Jemec GB	1998	Topical clindamycin versus systemic tetracycline in the treatment of hidradenitis suppurativa	*Journal of the American Academy of Dermatology*	198	RCT
18	C.-W. YU CA	1990	Hidradenitis suppurativa: a disease of follicular epithelium, rather than apocrine glands	*British Journal of Dermatology*	185	O
19	Esmann S	2011	Psychosocial impact of hidradenitis suppurativa: a qualitative study.	*Acta Dermato-Venereologica*	175	O
20	Zouboulis CC	2015	Hidradenitis suppurativa/acne inversa: criteria for diagnosis, severity assessment, classification and disease evaluation	*Dermatology*	171	G
21	Sartorius K	2003	Suggestions for uniform outcome variables when reporting treatment effects in hidradenitis suppurativa	*British Journal of Dermatology*	170	O
22	Shlyankevich	2014	Hidradenitis suppurativa is a systemic disease	*Journal of the*	169	O

	J		with substantial comorbidity burden: a chart-verified case-control analysis	*American Academy of Dermatology*		
23	Von der Werth JM	2000	The natural history of hidradenitis suppurativa	*Journal of the American Academy of Dermatology*	169	O
24	Wolk K	2011	Deficiency of IL-22 contributes to a chronic inflammatory disease: pathogenetic mechanisms in acne inversa	*The Journal of Immunology*	166	O
25	Mortimer PS	1985	A double-blind controlled cross-over trial of cyproterone acetate in females with hidradenitis suppurativa	*British Journal of Dermatology*	165	RCT
26	Clemmensen OJ	1983	Topical treatment of hidradenitis suppurativa with clindamycin	*International Journal of Dermatology*	165	RCT
27	Gener G	2009	Combination therapy with clindamycin and rifampicin for hidradenitis suppurativa: a series of 116 consecutive patients	*Dermatology*	164	O
28	Saunte DML	2017	Hidradenitis Suppurativa Advances in Diagnosis and Treatment	*JAMA*	159	R
29	Saunte DM	2015	Diagnostic delay in hidradenitis suppurativa is a global problem	*British Journal of Dermatology*	157	O
30	König A	1999	Cigarette smoking as a triggering factor of hidradenitis suppurativa	*Dermatology*	155	O
31	Onderdijk AJ	2013	Depression in patients with hidradenitis suppurativa	*Journal of the European Academy of Dermatology and Venereology*	153	O
32	Jemec GBE	1996	Histology of hidradenitis suppurativa	*Journal of the American Academy of Dermatology*	151	O
33	Zouboulis CC	2017	Development and validation of the International Hidradenitis Suppurativa Severity Score System (IHS4), a novel dynamic scoring system to assess HS severity	*British Journal of Dermatology*	149	O
34	Slade DE	2003	Hidradenitis suppurativa: pathogenesis and management	*British Journal of Plastic Surgery*	149	R

35	Garg A	2017	Sex-and age-adjusted population analysis of prevalence estimates for hidradenitis suppurativa in the United States	*JAMA Dermatology*	146	O
36	van der Zee HH	2012	Hidradenitis suppurativa: viewpoint on clinical phenotyping, pathogenesis and novel treatments	*Experimental Dermatology*	146	R
37	Rompel R	2000	Long-term results of wide surgical excision in 106 patients with hidradenitis suppurativa	*Dermatologic Surgery*	144	O
38	Ring HC	2017	The follicular skin microbiome in patients with hidradenitis suppurativa and healthy controls	*JAMA Dermatology*	142	O
39	Sabat R	2012	Increased prevalence of metabolic syndrome in patients with acne inversa	*PloS One*	141	O
40	Van Der Zee HH	2009	The effect of combined treatment with oral clindamycin and oral rifampicin in patients with hidradenitis suppurativa	*Dermatology*	140	O
41	Mendonça CO	2006	Clindamycin and rifampicin combination therapy for hidradenitis suppurativa	*British Journal of Dermatology*	138	O
42	Kelly G	2015	Dysregulated cytokine expression in lesional and nonlesional skin in hidradenitis suppurativa	*British Journal of Dermatology*	137	O
43	Matusiak Ł	2010	Hidradenitis suppurativa markedly decreases quality of life and professional activity	*Journal of the American Academy of Dermatology*	132	O
44	Prens E	2015	Pathophysiology of hidradenitis suppurativa: An update	*Journal of the American Academy of Dermatology*	129	R
45	Miller I	2011	A double-blind placebo-controlled randomized trial of adalimumab in the treatment of hidradenitis suppurativa	*British Journal of Dermatology*	128	RCT
46	Laffert M	2010	Hidradenitis suppurativa (acne inversa): early inflammatory events at terminal follicles and at interfollicular epidermis	*Experimental Dermatology*	128	O
47	Laffert M	2016	Safety and efficacy of anakinra in severe hidradenitis suppurativa: a randomized clinical trial	*JAMA Dermatology*	127	RCT
48	Martínez F	2001	Hidradenitis suppurativa and Crohn's disease: Response to treatment with infliximab	*Inflammatory Bowel Diseases*	125	O
49	A L Lima	2016	Keratinocytes and neutrophils are important sources of proinflammatory molecules in hidradenitis suppurativa	*British Journal of Dermatology*	124	O
50	Gregor B E	2015	Hidradenitis suppurativa: Epidemiology and	*Journal of the*	124	O

	Jemec		scope of the problem	*American Academy of Dermatology*		
51	Jemec GB	1988	The symptomatology of hidradenitis suppurativa in women	*British Journal of Dermatology*	124	O
52	Kimball AB	2014	Assessing the validity, responsiveness and meaningfulness of the Hidradenitis Suppurativa Clinical Response (HiSCR) as the clinical endpoint for hidradenitis suppurativa treatment	*British Journal of Dermatology*	122	O
53	Anne M R Schrader	2014	Hidradenitis suppurativa: A retrospective study of 846 Dutch patients to identify factors associated with disease severity	*Journal of the American Academy of Dermatology*	122	O
54	Florence Canoui-Poitrine	2013	Identification of Three Hidradenitis Suppurativa Phenotypes: Latent Class Analysis of a Cross-Sectional Study	*Journal of Investigative Dermatology*	122	O
55	Cusack C	2006	Etanercept: effective in the management of hidradenitis suppurativa	*British Journal of Dermatology*	122	O
56	Matusiak Ł	2009	Increased serum tumour necrosis factor-α in hidradenitis suppurativa patients: is there a basis for treatment with anti-tumour necrosis factor-α agents?	*Acta Dermato-Venereologica*	118	O
57	Ritz JP	1998	Extent of surgery and recurrence rate of hidradenitis suppurativa	*International Journal of Colorectal Disease*	118	O
58	Miller IM	2014	Association of metabolic syndrome and hidradenitis suppurativa	*JAMA Dermatology*	117	O
59	Gold DA	2014	The prevalence of metabolic syndrome in patients with hidradenitis suppurativa	*Journal of the American Academy of Dermatology*	116	O
60	Shavit E	2015	Psychiatric comorbidities in 3207 patients with hidradenitis suppurativa	*Journal of the European Academy of Dermatology and Venereology*	114	O
61	Marzano AV	2013	Pyogenic arthritis, pyoderma gangrenosum, acne, and hidradenitis suppurativa (PAPASH): a	*JAMA Dermatology*	113	O

			new autoinflammatory syndrome associated with a novel mutation of the PSTPIP1 gene			
62	Egeberg A	2016	Risk of major adverse cardiovascular events and all-cause mortality in patients with hidradenitis suppurativa	*JAMA Dermatology*	112	O
63	Sullivan TP	2003	Infliximab for hidradenitis suppurativa	*British Journal of Dermatology*	112	O
64	Cosmatos I	2013	Analysis of patient claims data to determine the prevalence of hidradenitis suppurativa in the United States	*Journal of the American Academy of Dermatology*	110	O
65	Boer J	1996	Hidradenitis suppurativa or acne inversa. A clinicopathological study of early lesions	*British Journal of Dermatology*	110	O
66	Harrison BJ	1987	Recurrence after surgical treatment of hidradenitis suppurativa	*British Medical Journal*	110	O
67	Blok JL	2016	Ustekinumab in hidradenitis suppurativa: clinical results and a search for potential biomarkers in serum	*British Journal of Dermatology*	109	O
68	Von Der Werth JM	2000	The clinical genetics of hidradenitis suppurativa revisited	*British Journal of Dermatology*	108	O
69	Pink AE	2013	γ-Secretase mutations in hidradenitis suppurativa: new insights into disease pathogenesis	*The Journal of Investigative Dermatology*	106	O
70	Lapins J	1999	Coagulase-negative staphylococci are the most common bacteria found in cultures from the deep portions of hidradenitis suppurativa lesions, as obtained by carbon dioxide laser surgery	*British Journal of Dermatology*	106	O
71	Mortimer PS	1986	Mediation of hidradenitis suppurativa by androgens	*British Medical Journal*	106	O
72	Van der Zee HH	2010	Deroofing: a tissue-saving surgical technique for the treatment of mild to moderate hidradenitis suppurativa lesions	*Journal of the American Academy of Dermatology*	104	O
73	Adams DR	2010	Treatment of hidradenitis suppurativa with etanercept injection	*Archives of Dermatology*	104	RCT

74	Verdolini R	2013	Metformin for the treatment of hidradenitis suppurativa: a little help along the way	*Journal of the European Academy of Dermatology and Venereology*	102	O
75	Van der Zee HH	2010	Hidradenitis suppurativa and inflammatory bowel disease: are they associated? Results of a pilot study	*British Journal of Dermatology*	102	O
76	Fardet L	2007	Infliximab for severe hidradenitis suppurativa: Transient clinical efficacy in 7 consecutive patients	*Journal of the American Academy of Dermatology*	101	O
77	Kromann CB	2014	Risk factors, clinical course and long-term prognosis in hidradenitis suppurativa: a cross-sectional study	*British Journal of Dermatology*	100	O
78	Attanoos	1995	The pathogenesis of hidradenitis suppurativa: a closer look at apocrine and apoeccrine glands	*British Journal of Dermatology*	100	O
79	Boer J	1999	Long-term results of isotretinoin in the treatment of 68 patients with hidradenitis suppurativa	*Journal of the American Academy of Dermatology*	98	O
80	Jemec GB	1996	Hidradenitis suppurativa-characteristics and consequences.	*Clinical and Experimental Dermatology*	98	O
81	Jemec GB	1996	The bacteriology of hidradenitis suppurativa	*Dermatology*	98	O
82	Fitzsimmons JS	1985	Evidence of genetic factors in hidradenitis suppurativa	*British Journal of Dermatology*	98	O
83	Barth JH	1996	Endocrine factors in pre- and postmenopausal women with hidradenitis suppurativa	*British Journal of Dermatology*	97	O
84	Shalom G	2015	Hidradenitis suppurativa and metabolic syndrome: a comparative cross-sectional study of 3207 patients	*British Journal of Dermatology*	96	O
85	KRoMAnn CB	2014	The influence of body weight on the prevalence and severity of hidradenitis suppurativa	*Acta Dermato-Venereologica*	96	O
86	M von Laffert	2011	Hidradenitis suppurativa/acne inversa: bilocated epithelial hyperplasia with very different sequelae	*British Journal of Dermatology*	96	O
87	Giamarellos-Bourboulis	2007	Altered innate and adaptive immune responses in patients with hidradenitis suppurativa	*British Journal of*	96	O

	EJ			*Dermatology*		
88	Alikhan A	2019	North American clinical management guidelines for hidradenitis suppurativa: A publication from the United States and Canadian Hidradenitis Suppurativa Foundations Part I: Diagnosis, evaluation, and the use of complementary and procedural management	*Journal of the American Academy of Dermatology*	95	O
89	Gulliver W	2017	Evidence-based approach to the treatment of hidradenitis suppurativa/acne inversa, based on the European guidelines for hidradenitis suppurativa	*Reviews in Endocrine and Metabolic Disorders*	94	G
90	Wortsman X	2014	Ultrasound in-depth characterization and staging of hidradenitis suppurativa	*Dermatologic Surgery*	94	O
91	Sellheyer K	2005	"Hidradenitis suppurativa" is acne inversa! An appeal to (finally) abandon a misnomer	*International Journal of Dermatology*	94	R
92	Sabat R	2020	Hidradenitis suppurativa	*Nature Reviews. Disease Primers*	93	R
93	Alavi A	2015	Quality-of-life impairment in patients with hidradenitis suppurativa: a Canadian study	*American Journal of Clinical Dermatology*	93	O
94	Vinding GR	2014	The prevalence of inverse recurrent suppuration: a population- based study of possible hidradenitis suppurativa	*British Journal of Dermatology*	93	O
95	Van der Zee HH	2012	Alterations in leucocyte subsets and histomorphology in normal-appearing perilesional skin and early and chronic hidradenitis suppurativa lesions	*British Journal of Dermatology*	92	O
96	Ingram JR	2018	Population-based Clinical Practice Research Datalink study using algorithm modelling to identify the true burden of hidradenitis suppurativa	*British Journal of Dermatology*	92	O
97	Lapins J	2001	Incidence of cancer among patients with hidradenitis suppurativa	*Archives of Dermatology*	92	O
98	Napolitano M	2017	Hidradenitis suppurativa: from pathogenesis to diagnosis and treatment	*Clinical, Cosmetic and Investigational Dermatology*	91	R
99	Giamarellos-Bourboulis	2008	An open-label phase II study of the safety and efficacy of etanercept for the therapy of	*British Journal of*	90	O

	EJ		hidradenitis suppurativa	*Dermatology*		
100	Kohorst JJ	2015	Systemic associations of hidradenitis suppurativa	*Journal of the American Academy of Dermatology*	89	R
